# Characterization of Major Cell-Wall-Degrading Enzymes Secreted by *Diaporthe* spp. Isolate Z1-1N Causing Postharvest Fruit Rot in Kiwifruit in China

**DOI:** 10.3390/biology13121006

**Published:** 2024-12-02

**Authors:** Li-Zhen Ling, Ling-Ling Chen, Jia-Yu Ma, Chao-Yue Li, Dong-Ru Zhang, Xiao-Di Hu, Shu-Dong Zhang

**Affiliations:** 1 Key Laboratory City for Study and Utilization of Ethnic Medicinal Plant Resources of Western Guizhou Province, Liupanshui Normal University, Liupanshui 553004, China; primula-ling@163.com (L.-Z.L.); c.l-04.30@foxmail.com (L.-L.C.); yamiyu0604@foxmail.com (J.-Y.M.); tf-lmuiaer@foxmail.com (C.-Y.L.); zhdongru@126.com (D.-R.Z.); 2 State Key Laboratory for Managing Biotic and Chemical Threats to the Quality and Safety of Agro-Products, Key Laboratory of Biotechnology in Plant Protection of MARA, Key Laboratory of Green Plant Protection of Zhejiang Province, Institute of Plant Virology, Ningbo University, Ningbo 315211, China

**Keywords:** kiwifruit, cell-wall-degrading enzymes, *Diaporthe*, pathogenicity

## Abstract

Pathogen-induced fruit decay is a significant threat to the kiwifruit industry, leading to considerable economic losses annually. The cell-wall-degrading enzymes (CWDEs) secreted by these pathogens are crucial for penetrating the cell wall and accessing nutrients. Among them, *Diaporthe* species are recognized as major causal agents of soft rot in kiwifruit, yet their pathogenic mechanisms are not well understood. In this study, we explored the production of various CWDEs secreted by *Diaporthe* Z1-1N, including polygalacturonase (PG), polymethylgalacturonase (PMG), polygalacturonic acid transeliminase (PGTE), pectin methyltranseliminase (PMTE), endoglucanase (Cx), and β-glucosidase (β-glu), both in liquid cultures and within infected kiwifruit tissues. Our findings revealed significant activities of two pectinases (PG and PMG) and cellulases (Cx and β-glu) in the infected tissues. In contrast, very low levels of PMTE and PGTE activities were observed under the same conditions. When orange pectin served as the carbon source, PG and PMG showed notable activities, while PMTE and PGTE remained inactive. Moreover, the activities of Cx and β-glu significantly decreased by more than 63 times in the liquid medium with carboxymethyl cellulose (CMC) as the carbon source compared to their levels in infected kiwifruit. A further analysis indicated that the necrotic lesions produced by pectinase extracts were larger than those produced by cellulase extracts. Notably, four enzymes—PG, PMG, Cx, and β-glu—exhibited high activities on the third or fourth day post-infection with *Diaporthe* Z1-1N. These results suggest that *Diaporthe* Z1-1N secretes a range of CWDEs that contribute to kiwifruit decay by enhancing the activities of PG, PMG, Cx, and β-glu. This study sheds light on the pathogenicity of *Diaporthe* in kiwifruit and highlights the importance of these enzymes in the decay process.

## Simple Summary

To better understand the pathogenicity of *Diaporthe isolate* Z1-1N, one of the major causal agents of soft rot disease in kiwifruit, we detected the activities of six CWDEs, including polygalacturonase (PG), polymethylgalacturonase (PMG), polygalacturonic acid transeliminase (PGTE), pectin methyltranseliminase (PMTE), endoglucanase (Cx), and β-glucosidase (β-glu), in vitro and in vivo. Our results indicated that *Diaporthe* Z1-1N can secrete four pectinases and two cellulases, which exhibited varying degrees of pathogenicity. Among the four pectinases, PG and PMG consistently demonstrated high activities in both liquid culture media and infected tissues. In contrast, only low activities of PGTE and PMTE were observed during infection, with no activity detected in the liquid cultures. Additionally, two cellulases of β-glucosidase and endoglucanase exhibited differing levels of activities, particularly showing a significant increase during infection by *Diaporthe* Z1-1N. Our results indicated that the activities of PG, PMG, β-glu, and Cx peaked at three or four days after infection with *Diaporthe* Z1-1N on kiwifruit. Furthermore, the levels of PG and PMG were higher than those of Cx and β-glu throughout the incubation periods. In conclusion, *Diaporthe* Z1-1N can secrete various types of pectinases and cellulases, with cellulases (Cx and β-glu) and pectinases (PMG and PG) playing crucial roles during infection.

## 1. Introduction

Kiwifruit (*Actinidia* spp.) is a deciduous vine with approximately 54 identified species and 75 taxa worldwide [1]. The fruit of kiwifruit has a rich nutritional profile, including vitamins, minerals, dietary fiber, phenols, carotenoids, and other essential nutrients [2,3,4,5]. Additionally, its sweetness and flavor have made it a favorite among consumers. However, soft rot disease poses a significant threat to kiwifruit quality during storage. Studies have reported that the fungi from *Botryosphaeria dothidea* and *Diaporthe* spp. are the primary culprits responsible for kiwifruit soft rot disease [6,7,8], which affects 20–50% of the harvested fruits during post-harvest handling and storage and leads to substantial annual economic losses [9].

Plants have evolved effective resistance mechanisms to fend off pathogen attacks. A primary challenge for pathogens is penetrating the host cell wall, the fundamental physical barrier that protects plants from microbial invasion [10]. The plant cell wall is composed of polysaccharides, including cellulose, hemicellulose, and pectin [11]. Most plant pathogenic fungi secrete a diverse array of cell-wall-degrading enzymes (CWDEs), which are crucial for infection as they degrade these cell wall components to access nutrients [12,13]. Studies have shown that enzymes like pectinase and cellulase, including polymethyl-galacturonase (PMG), endoglucanase (Cx), β-glucosidase (β-glu), pectin methylesterase (PME), β-galactosidase (β-gal), polygalacturonase (PG), and pectate lyase (PL), play significant roles in the pathogenicity of various pathogens [13,14,15,16,17].

Two pectinases, PMG and PG, hydrolyze pectate and pectin by cleaving the α-1,4-glycosidic bonds in the D-galacturonic acid moieties of pectic substances, facilitated by the introduction of water across the oxygen bridge [13,17]. Cellulases (Cxs) are a group of enzymes including endoglucanase (Cx; EC 3.2.1.4), cellobiohydrolase (C1; EC 3.2.1.91), and β-glu (EC 3.2.1.21). Cx degrades cellulose into cellobiose, with β-glu further cleaving it into glucose monomers [17]. These enzymes exhibit high activities in liquid culture and in host tissues inoculated with various fungal pathogens, and they have been identified as the virulence factors [14,15,16,17]. Additionally, polygalacturonic acid transeliminase (PGTE) and pectin methyltranseliminase (PMTE) are forms of pectate lyase (PL) that cleave the α-1,4-linkage between methylgalacturonides in the pectin molecule and eliminate the hydrogen at C5. PGTE specifically cleaves the α-1,4-glycosidic bond within the pectinate molecule, whereas PMTE targets pectin or methylated polygalacturonic acid in the cell wall [18,19].

Species of *Diaporthe* and their *Phomopsis* asexual states are plant pathogens and non-pathogenic endophytes with broad host ranges and a global distribution [20]. MycoBank (https://www.mycobank.org/Simple%20names%20search, accessed on 20 October 2024) lists over 1000 species within the genus *Phomopsis*, while the genus *Diaporthe* encompasses more than 1300 taxa. Several plant pathogenic *Diaporthe* species are associated with cankers, diebacks, rots, spots, and wilts across a diverse array of plants [21]. The sequenced genomes of the *P. longicolla* isolate MSPL 10-6 and *Diaporthe eres* isolate P3-1W have revealed a significant number of genes encoding CWDEs, identified through computational biology approaches [22,23]. Our previous studies indicated that the isolate *Diaporthe* spp. Z1-1N exhibited strong pathogenicity and acted as a causative agent of kiwifruit soft rot disease [24]. However, the role of secretory enzymes produced by *Diaporthe* spp. Z1-1N in relation to soft rot has yet to be characterized. Consequently, this study aims to establish the role of CWDEs from *Diaporthe* spp. Z1-1N in the pathogenicity associated with kiwifruit soft rot disease.

## 2. Materials and Methods

### 2.1. Plant Material and Pathogenic Fungi

Kiwifruits (*A. chinensis* cv. Hongyang) of similar size were purchased from the local market. The isolate of *Diaporthe* spp. Z1-1N was provided by the School of Biological Science and Technology at Liupanshui Normal University. This isolate was cultured on potato dextrose agar (PDA) medium at 25 °C in the dark for 6 days. It was then used to obtain inoculum for the enzyme assays and pathogenicity trials in this study.

### 2.2. Enzyme Production in Liquid Culture

In this study, a modified Marcus liquid medium was employed, which contained KNO_3_ (2 gL^−1^), KH_2_PO_4_ (1 gL^−1^), MgSO_4_·7H_2_O (0.5 gL^−1^), FeSO_4_ (0.01 gL^−1^), KCl (0.5 gL^−1^), VB_1_ 0.02 mg, and L-asparagine (0.5 gL^−1^). For quantitative estimation, mycelial discs (5 mm) from a 6-day-old PDA culture were inoculated into the liquid medium (pH 5.0), utilizing orange pectin and CMC as carbon sources (1% *w*/*v*) for the production of pectinase and cellulase, respectively. A total of 100 mL of liquid medium was dispensed into 250 mL flasks and placed on an orbital shaker set at 100 rpm and 28 °C. Cultures were harvested on the third day, and the contents were filtered using vacuum to remove the fungal mycelia. The filtrates were then centrifuged at 10,000× *g* for 15 min at 4 °C, and the supernatant was utilized to assess the enzyme activity.

### 2.3. Fruit Infection and Crude Enzyme Extraction

A 5 mm inoculum was utilized for the infection experiments conducted on kiwifruit, as previously described [23]. The isolate of *Diaporthe* Z1-1N was inoculated onto PDA medium at 25 °C. A 5 mm diameter PDA plug was extracted using a punch on the third day of incubation. Healthy ‘Hongyang’ kiwifruit was surface-sterilized and pricked with an inoculation needle to a depth of 2–3 mm. The mycelial surface of the 5 mm plug was placed onto the fruit wound, which was then sealed in a vessel at 28 °C for regular observation of disease development. The inoculated fruits were also treated with a sterile PDA plug as a control. Three biological replicates were established for each treatment.

Rotten tissues were collected at 0.5 days post-infection (dpi) and from 1 to 6 dpi to examine enzyme activity. Enzyme extraction followed the procedures outlined in our previous work [23]. Briefly, 3 g of frozen samples was ground with 12 mL of 2 M sodium chloride buffer solution, which contained 10 mmol/L EDTA and 5 g/L PVP adjusted to pH of 7.4 using 0.01 mol L^−1^ NaOH. The resulting homogeneous solution was centrifuged at 15,000× *g* for 30 min at 4 °C. The supernatant was collected and used as the crude enzymatic extract to measure the activities of PMG, PG, PGTE, PMTE, β-glu, and Cx.

### 2.4. Enzymic Activity Assays

The activities of PMG, PG, β-glu, and Cx were determined using the 3,5-dinitrosalicylic acid (DNS) colorimetric method, following the procedures outlined by Xue and Ling with minor modifications [17,23]. For the determination of PMG and PG activities, the reaction mixture consisted of 0.5 mL of crude enzyme extract, 1.0 mL of Na-acetate buffer (50 mM, pH 4.4) and 0.5 mL substrate, which was incubated for 60 min at 37 °C. The reaction was terminated by the addition of 3 mL of DNS by boiling for 10 min, after which the absorbance was measured at 540 nm. The substrates used for PG and PMG were 1.0% (*m*/*v*) polygalacturonic acid and 1.0% (*m*/*v*) orange pectin, respectively. Galacturonic acid at various concentrations was employed to establish a standard curve. For Cx and β-glu activities, 1.0 mL of crude enzyme was mixed with 1 mL of substrate in acetate buffer (0.05 M, pH 5.5) and incubated for 30 min at 37 °C. Subsequently, 3 mL of DNS was added to the reaction mixture, which was then boiled for 10 min. The OD values of the reducing products were measured at 540 nm after cooling. The substrates for Cx and β-glu were 1% (*w*/*v*) carboxymethyl cellulose, and 1% *m*/*v* salicin, respectively. Glucose at various concentrations was used to establish a standard curve.

The activities of PGTE and PMTE were measured using the method described by Ge et al. [19]. A crude enzyme solution (100 μL) was combined with 4.0 mL of Gly-NaOH (50 mM, pH 9.0) and 1.0 mL of 3 mM CaCl_2_ in a reaction mixture containing 0.3 mL of 1 mg·mL^−1^ substrate (polygalacturonic acid for PGTE and pectin for PMTE), and the mixture was incubated at 30 °C for 5 min. Absorbance was subsequently measured at 232 nm. Protein concentrations were determined using Bradford’s method [25], with bovine serum albumin serving as the standard for the establishment of a standard curve. The activities of PGTE and PMTE were expressed in units (U), where 1 U is defined as the release of 1 μg of unsaturated galacturonic acid per mg of protein per minute.

### 2.5. Purification of Crude Enzyme Extracts and Inoculation on Kiwifruit Fruit

Pectinase and cellulase from the liquid culture of *Diaporthe* isolate Z1-1N were separately induced for production using orange pectin and CMC as carbon resources. The enzymes were purified following the procedure outlined by Zhang with minor modifications [26]. One hundred mL of crude enzyme extracts of pectinase and cellulase were subjected to 80% ammonium sulfate precipitation under cold conditions. The resulting precipitate was collected via centrifugation at 10,000× *g* for 30 min at 4 °C, then dissolved in 10 mL of sodium acetate buffer (0.05 M, pH 5.0) containing 1 M NaCl and 1 mM EDTA. This solution was subsequently dialyzed for 24 h at 4 °C with continuous stirring and the buffer was changed twice. The dialyzed sample was finally stored at −20 °C for future use.

To assess the pathogenic potential of these enzymes, 0.5 mL of dialyzed samples of pectinase and cellulase were separately applied to the surface of fresh healthy kiwifruits, which had been punctured with 4–5 pinholes. These fruits, enclosed in sealed vessels, were then incubated at 25 °C in darkness until symptomatic tissues appeared. An equivalent volume of sterile water served as the control. To compare the symptoms induced by crude enzyme extracts with those caused by pathogens, a 5 mm mycelial plug obtained from 72 h old cultures of the *Diaporthe* isolate Z1-1N, grown on PDA at 25 °C, was placed on the adaxial surface of the fruit, with the mycelial side facing the fruit, and incubated under the same conditions. Sterile PDA plugs were utilized as controls. Three biological trials were conducted, each consisting of three fruits.

### 2.6. Statistical Analysis

All experiments were repeated three times, and data were collected accordingly. The mean and standard error were calculated from the acquired data. Differences among the means of the values were analyzed using one-way analysis of variance (ANOVA) with SPSS 19.0 for Windows (SPSS Inc., Chicago, IL, USA). Mean separations were conducted using Duncan’s multiple range test, with differences considered statistically significant at *p* < 0.05.

## 3. Results

### 3.1. Production of Cell-Wall-Degrading Enzymes In Vitro Cultures and Infected Tissues

In this study, we detected the activities of six enzymes, including PGTE, PMTE, Cx, β-glu, PMG, and PG, in in vitro cultures. Our results demonstrated that only PG and PMG exhibited high activities after three days of incubation in the medium containing pectin as the carbon source. Their activities had no significant differences and were 9.05 U/mL for PMG and 9.04 U/mL for PG, respectively (Table 1). In contrast, no activities of PGTE or PMTE were detectable in the pectin-containing medium (Table 1). When CMC was used as the carbon source, only traces of Cx and β-glu activities were detected during cultivation, both measuring 0.38 U/mL (Table 1). Previous studies have indicated that these six enzymes, particularly Cx, β-glu, PMG, and PG, exhibit activities during fungal infections [27,28]. In the present study, we also detected their activities in rotted tissues infected by *Diaporthe* Z1-1N at 3 dpi. The results indicated that PMG exhibited the highest activity (29.83 U/mL) followed by PG (27.85 U/mL) (Table 1). Additionally, low activity levels of PGTE and PMTE were detected, measuring 0.45 U/mL and 0.38 U/mL, respectively (Table 1). Notably, the activities of Cx and β-glu showed significant increases and were 63.9 and 63.6 times higher than those in the medium containing CMC as the carbon source, respectively (Table 1). However, the activities of Cx and β-glu were still significantly lower than those of PG and PMG (Table 1). Numerous studies have reported that plant fungal pathogens produce Cx, β-glu, PMG, and PG in lipid cultivation and in infected host tissues [14,17]. Consistent with these findings, our study also yielded similar results. Collectively, these results suggest that four enzymes (Cx, β-glu, PMG, and PG) can be secreted by *Diaporthe* Z1-1N. Furthermore, their high activities in infected host tissue imply that these enzymes may play a crucial role in the pathogenicity of *Diaporthe* Z1-1N. In contrast, only trace amounts of PGTE and PMTE activities were measured in the rotted fruit tissues. We infer that these two enzymes may be involved in the process of disease establishment by this fungal pathogen.

### 3.2. Effects of the Enzyme Extracts of Pectinase and Cellulase on Kiwifruit Fruit

**Figure 1:**
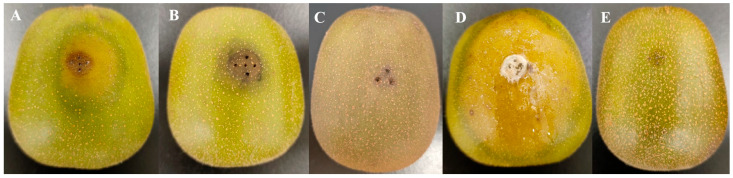
Effects of crude enzyme extract from *Diaporthe* Z1-1N liquid culture and the mycelium inoculated in fruit for 7 days. (**A**) Crude pectinase extract inoculation; (**B**) Crude cellulase extract inoculation; (**C**) The sterile water; (**D**) Mycelial plug inoculation; (**E**) Sterile potato dextrose agar plug inoculation.

In this study, we investigated the effects of the crude enzyme extracted from a lipid culture filtrate on kiwifruit tissue. Pectinase and cellulase were prepared from a three-day-old shaken culture using orange pectin and CMC as the sole carbon sources, respectively. When these enzyme extracts were applied to kiwifruit fruits, we observed that both crude enzymatic extracts of pectinase and cellulase induced symptoms of necrotic lesions (Figure 1A,B), whereas the distilled water did not produce any symptoms of necrotic lesions (Figure 1C). Notably, the necrotic lesions induced by the cellulase extracts were smaller than those produced by the pectinase extracts (Figure 1A,B). Considering the low activity levels of the cellulases (Cx and β-glu) and the high activity levels of the pectinases (PMG and PG) (Table 1), we inferred that the observed differences in necrotic lesions might be related to their respective activities in liquid cultures. Furthermore, our results demonstrated that the symptoms developed by both crude enzyme extracts were similar but mild compared to those caused by infection with *Diaporthe* Z1-1N mycelium (Figure 1D). In contrast, the control fruits inoculated with sterile PDA plugs exhibited no lesions (Figure 1E). Therefore, these findings suggest that both pectinase and cellulase are indeed involved in lesion development; however, they may play a part in *Diaporthe* Z1-1N disease progression.

### 3.3. Changes in Cell-Wall-Degrading Enzymes During Infection of Diaporthe Z1-1N

**Figure 2:**
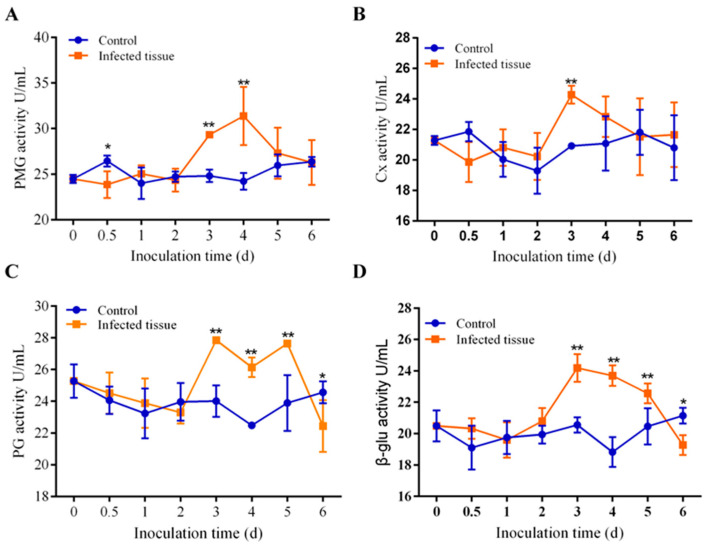
Effects of *Diaporthe* Z1-1N infection on activities of (**A**) PMG, Cx (**B**), PG (**C**), and β-glu (**D**) in rotted kiwifrui fruit. The asterisks indicate significant difference between control and *Diaporthe* Z1-1N-inoculated fruit (* *p* < 0.05, ** *p* < 0.01).

To further understand the roles of CWDEs during disease development, we measured the activities of Cx, β-glu, PMG, and PG in infected tissues at various incubation periods of *Diaporthe* Z1-1N in this study. Our results indicated that the activities of these four enzymes showed no significant differences during the mock infection. However, when the fruit was incubated with *Diaporthe* Z1-1N, the activities of these enzymes were higher than those observed in lipid cultures. Additionally, the enzymes exhibited varying activities during different stages of the same infection (Figure 2A–D). Specifically, the PMG activity significantly increased at 3 dpi, peaked at 4 dpi, subsequently decreased during the fungal infection, and did not change after 5 dpi (Figure 2A). Notably, the PMG activity exhibited a significant decrease at 12 h post-infection with *Diaporthe* Z1-1N, which contrasted with the behavior of the other three enzymes (Figure 2A–D). The Cx activity demonstrated a similar pattern, significantly increasing only at 3 dpi (Figure 2B). In addition, the PG and β-glu activities exhibited a significant increase from 3 dpi to 5 dpi, followed by a decrease at 6 dpi (Figure 2C,D). These results suggest that the four enzymes were evidently induced by *Diaporthe* Z1-1N, potentially playing distinct roles during disease development.

## 4. Discussion

Many phytopathogenic microorganisms, primarily fungi, have been detected on kiwifruit [29,30,31,32]. Species from the *Diaporthe* genus represent some of the most harmful fungal pathogens, capable of causing various diseases, such as stem canker, leaf spot blight, and fruit decay, which significantly impact kiwifruit yield and quality [33,34,35,36]. Currently, over 10 *Diaporthe* species are known to cause soft rot disease, resulting in considerable economic damage during the growing season and post-harvest storage [33,36,37,38,39,40,41]. However, there remains a lack of information regarding the pathogenicity of *Diaporthe* species in relation to kiwifruit fruit decay, an area that warrants further exploration due to their potential role in agriculture in preventing the propagation of pathogens. Many phytopathogenic fungi can secrete CWDEs to degrade plant cell wall components, thereby utilizing them as nutrients to colonize and propagate [12,13]. Consequently, the main objective of this study is to characterize several CWDEs (including PMG, Cx, PGTE, PMTE, PG, and β-glu) secreted by *Diaporthe* Z1-1N, assess the pathogenicity of these CWDEs, and investigate the changes in CWDE activity during fungal infections.

Our results indicated that four enzymes—PMG, Cx, PG, and β-glu—were detected in both in vitro cultures and during in vivo incubation. Previous studies have revealed that many phytopathogenic fungi can secrete these four enzymes [17,42,43,44]. For example, Xue et al. (2018) showed that *Rhizoctonia solani* causes peanut sheath blight by producing PG, PMG, β-glu, and Cx in the stalk and leaves of Baisha and Silihong peanut cultivars [17]. Moreover, studies have demonstrated that mutants of the genes encoding these enzymes exhibit significant functions [45,46]. Therefore, these four enzymes may be universal and important for the pathogenicity of phytopathogenic fungi. Additionally, we found that the activities of Cx and β-glu during in vivo *i*ncubation were higher than those observed in the in vitro culture. In contrast, the activities of PG and PMG increased three times more under in vivo conditions than in vitro conditions. This phenomenon was also noted in the tomato pathogen *Helminthosporium carposaprum* [43]. Consequently, the results indicated that the activities of these four enzymes were induced by *Diaporthe* Z1-1N. However, the inducing conditions for cellulases were more stringent than those for pectinases, which could lead to their weaker pathogenicity in kiwifruit due to their lower activity levels. This study has demonstrated that the activities of PG and PMG were higher than those of Cx and β-glu, which appeared in the later stages of the degradation process [47]. In the present study, the levels of PG and PMG were indeed higher than those of Cx and β-glu during the incubation process. However, these enzymes were significantly activated at 3 dpi. Indeed, these enzymes are involved in initial tissue maceration and the degradation of cell wall materials. An earlier study from our laboratory demonstrated that the activities of PME and β-gla peaked at 2 dpi for *Diaporthe* P3-1W on kiwifruit [23]. Whether *Diaporthe* Z1-1N also produces these enzymes requires further investigation.

In contrast, low activity levels of PGTE and PMTE were detected only during in vivo incubation. A possible explanation for this is that the activities of two enzymes are below the detection threshold in the in vitro culture. Previous studies have indicated that pectinase activity levels are influenced by factors such as Ca^2+^, pH levels, and the culture medium [43,48]. Therefore, the culture conditions for these two enzymes will be further optimized in liquid cultures. Additionally, several studies have demonstrated that PL secreted by pathogens is involved in soft rot in various plants [17,19]. Genome data from several *Diaporthe* species have also identified the genes encoding these enzymes [23,40]. Whether these enzymes contribute to pathogenicity requires further investigation. Collectively, these results suggest that *Diaporthe* Z1-1N can secrete different types of pectinase and cellulase. Furthermore, cellulases (Cx and β-glu) and pectinases (PMG and PG) play a crucial role in the fungal infection process of kiwifruit.

## 5. Conclusions

In this study, *Diaporthe* Z1-1N was found to secrete a range of CWDEs, including four pectinases and two cellulases, and exhibited varying degrees of pathogenicity. Among the four pectinases, PG and PMG consistently demonstrated high levels of activity in both the liquid culture medium and infected kiwifruit tissues. In contrast, only low activities of PGTE and PMTE were observed during kiwifruit infection, with no activities detected in the liquid culture. Additionally, two cellulases, β-glu and Cx, exhibited differing activity levels, particularly showing a significant increase during kiwifruit infection by *Diaporthe* Z1-1N. Our results indicated that the activities of PG, PMG, β-glu, and Cx peaked at 3 or 4 dpi of *Diaporthe* Z1-1N on kiwifruit. Furthermore, the levels of PG and PMG were higher than those of Cx and β-glu throughout the incubation periods. In conclusion, *Diaporthe* Z1-1N can secrete various types of pectinases and cellulases, with cellulases (Cx and β-glu) and pectinases (PMG and PG) playing a crucial role in the fungal infection process.

## Figures and Tables

**Table 1:** Activity of cell-wall-degrading enzymes including polygalacturonase (PG), polymethylgalacturonase (PMG), cellulase (Cx), and β-glucosidase in liquid culture and rotted kiwifruit tissue infected by *Diaporthe* Z1-1N.

Type	Enzyme	Enzymatic Activity (U/mL)	
		In Vitro Culture	In Vivo Incubation
Pectinase	PMG	9.05 ± 0.27 a	29.33 ± 0.26 a
	PG	9.04 ± 0.42 a	27.85 ± 0.22 b
	PMTE	/	37.60 × 10^−2^ ± 12.44 × 10^−2^ d
	PGTE	/	44.55 × 10^−2^ ± 6.95 × 10^−2^ d
Cellulase	Cx	38.28 × 10^−2^ ± 2.32 × 10^−2^ b	24.29 ± 0.59 c
	β-Glu	38.13 × 10^−2^ ± 0.13 × 10^−2^ b	24.18 ± 0.88 c

Note: Values are the mean of three replicates ± SD. Different letters in each column indicate significant differences (*p* < 0.05).

## Data Availability

Data are contained within the article.
